# Beyond 2D telestration: an evaluation of novel proctoring tools for robot-assisted minimally invasive surgery

**DOI:** 10.1007/s11701-016-0564-1

**Published:** 2016-02-25

**Authors:** Anthony M. Jarc, Swar H. Shah, Troy Adebar, Eric Hwang, Monish Aron, Inderbir S. Gill, Andrew J. Hung

**Affiliations:** Medical Research, Intuitive Surgical, Inc, Norcross, GA USA; Department of Mechanical Engineering, Stanford University, Stanford, CA USA; Department of Urology, USC Institute of Urology, Keck School of Medicine, University of Southern California, Los Angeles, CA USA

**Keywords:** Robot-assisted surgery, Proctoring, Tele-mentoring, Training

## Abstract

Experienced surgeons commonly mentor trainees as they move through their initial learning curves. During robot-assisted minimally invasive surgery, several tools exist to facilitate proctored cases, such as two-dimensional telestration and a dual surgeon console. The purpose of this study was to evaluate the utility and efficiency of three, novel proctoring tools for robot-assisted minimally invasive surgery, and to compare them to existing proctoring tools. Twenty-six proctor-trainee pairs completed validated, dry-lab training exercises using standard two-dimensional telestration and three, new three-dimensional proctoring tools called ghost tools. During each exercise, proctors mentored trainees by correcting trainee technical errors. Proctors and trainees completed post-study questionnaires to compare the effectiveness of the proctoring tools. Proctors and trainees consistently rated the ghost tools as effective proctoring tools. Both proctors and trainees preferred 3DInstruments and 3DHands over standard two-dimensional telestration (proctors *p* < 0.001 and *p* = 0.03, respectively, and trainees *p* < 0.001 and *p* = 0.002, respectively). In addition, proctors preferred three-dimensional vision of the operative field (used with ghost tools) over two-dimensional vision (*p* < 0.001). Total mentoring time and number of instructions provided by the proctor were comparable between all proctoring tools (*p* > 0.05). In summary, ghost tools and three-dimensional vision were preferred over standard two-dimensional telestration and two-dimensional vision, respectively, by both proctors and trainees. Proctoring tools—such as ghost tools—have the potential to improve surgeon training by enabling new interactions between a proctor and trainee.

## Introduction

As robot-assisted minimally invasive surgery (RAMIS) continues to expand into new surgical specialties, it is important to efficiently guide new surgeons through their learning curves to maximize patient safety [1–3]. One common element of new surgeon training pathways is proctored cases, where an experienced surgeon mentors a trainee [4, 5]. In RAMIS, this typically occurs during the first series of cases undertaken by a surgeon or during complex cases where experienced surgeon input could be helpful.

The current standard of RAMIS proctoring is in-person proctoring using two-dimensional (2D) telestration on the vision cart touchscreen. A more expensive alternative is a dual surgeon console that also allows a proctor to use three-dimensional (3D) pointers to provide instruction to a training surgeon. Given the time and geographic constraints of proctors, researchers have explored a remote proctoring technology for da Vinci^®^ Surgical Systems (Intuitive Surgical, Inc., Sunnyvale, CA, USA) called da Vinci Connect™ [6–9]. da Vinci Connect enables a proctor to remotely view a surgeon’s operative field on his laptop, provide verbal instruction, and telestrate using a mouse or a laptop’s touchscreen. Researchers have found that remote proctoring using da Vinci Connect was feasible and effective [6, 9].

Whether local or remote, the types of interactions between proctors and trainees can be extended beyond 2D telestration and a dual surgeon console given the architecture of RAMIS systems. For example, the proctor might be able to better visualize the operative field using a 3D view [10–17], similar to the surgeon console but using a low-cost, remote setup. Furthermore, given a 3D display, a proctor could interact with the trainee in 3D in new ways. For example, a proctor can explicitly demonstrate how to position the instruments in the operative field and ask the trainee to match postures rather than trying to verbally explain the configuration or draw the configuration in 2D. As with any advanced interaction, these tools need to help with instruction for the trainee, yet not be cumbersome or frustrating for the proctor. Therefore, they must be extensively studied from both the proctor’s and trainee’s perspectives to ensure the appropriate interactions are delivered.

In this study, we examine the utility and efficiency of three novel, 3D proctoring tools, in the form of semi-transparent ghost tools overlaid on the surgeon’s field of view, and compare them to standard 2D telestration. We hypothesized that the 3D ghost tools would enable proctors to more effectively mentor trainees and enable trainees to more effectively extract meaning from proctor input.

## Methods

### Ghost tools setup

**Fig. 1:**
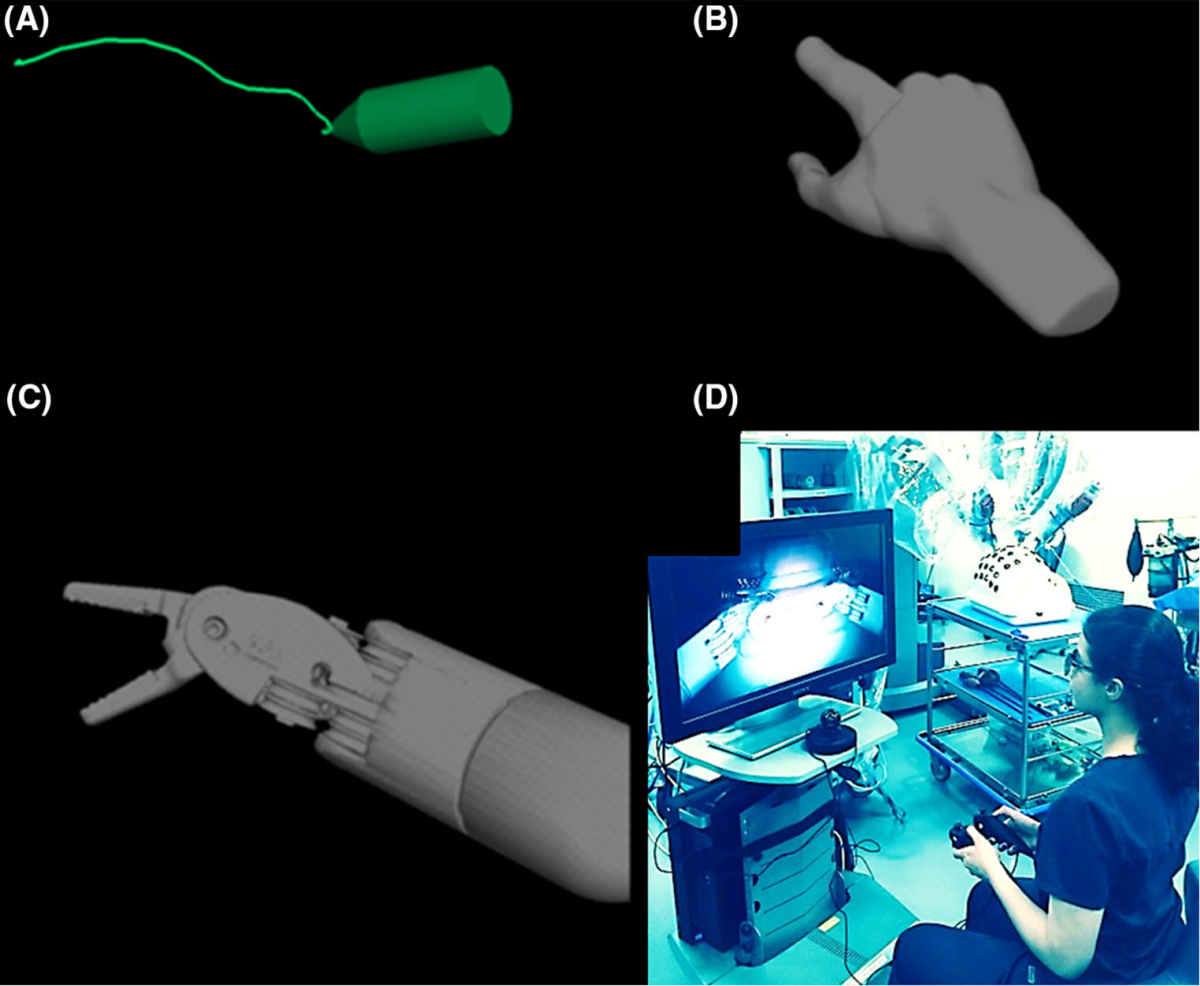
The three versions of ghost tools: **a** 3DPointers, **b** 3DHands, **c** 3DInstruments and the experimental setup where a proctor controls the position, orientation, and state of ghost tools using a *Razer Hydra* and a 3D display

Conventional 2D telestration (2DTele) and three different types of 3D ghost tools—3D pointers (3DPointers), 3D cartoon hands (3DHands), and 3D instruments (3DInstruments)—were compared using the da Vinci Si™ Surgical System (Fig. 1). Custom software was written and run on an external PC to render the ghost tools as semi-transparent overlays on the stereoscopic, endoscopic image captured from the video output channels using Decklink Quad frame grabbers (Blackmagic Design Pty. Ltd., Fremont, CA, USA). The stereoscopic image with the ghost tools overlay was output from the PC and displayed to the trainee at the surgeon console in a sub-window using the 3D TilePro™ Display video inputs and to the proctor using a polarized 3D display (Sony, Inc., Fig. 1d). Importantly, similar setups as the one used in this study, which used readily available video input and output channels, can be replicated by other academic researchers to explore advanced proctoring tools on clinical da Vinci Surgical Systems.

The 3DPointers enabled proctors to point and draw in 3D. The 3DHands enabled proctors to position and orient a cartoon hand in 3D space as well as open and close their index fingers and thumbs to illustrate grasping objects. Finally, the 3DInstruments behaved similar to real da Vinci Endowrist^®^ Large Needle Driver instruments and were able to be positioned and oriented in 3D space while also opening and closing the instrument jaws to illustrate grasping objects. All three variations of ghost tools were controlled using Razer™ Hydra motion controllers (Sixense Entertainment, Inc., Los Gatos, CA, USA).

### User study

The effectiveness of the four proctoring tools (the three types of ghost tools and standard 2DTele) was examined during four, validated dry-lab exercises. The four dry-lab exercises were previously shown to have construct validity and included Ring Rollercoaster 4, Big Dipper, Around-the-World, and Figure-of-Eight Knot Tying on a luminal closure model (see Fig. 1 in [18] for task images) [18–20]. One proctor was randomly paired with one trainee to evaluate the four proctoring tools across the four exercises. Proctors were experienced surgeons (>50 cases) or experienced RAMIS trainers (>100 surgeons trained). Trainees included surgeons in training with RAMIS technology and new RAMIS trainers with limited exposure to RAMIS. The dry-lab exercises targeted technical skills related to using the da Vinci Surgical System as opposed to cognitive skills requiring surgical judgment in order to standardize across surgical and non-surgical proctors and trainees (see Table 1 in [18] for each exercise’s technical skills).

Each proctor-trainee pair completed the four exercises in random order with a proctoring tool randomly assigned to each exercise. Randomization was achieved by performing two paired random permutations for each subject: one for the four exercises and one for the four proctoring tools.

Informed consent was obtained from all individual participants included in the study (Western IRB, Puyallup, WA). Before beginning the exercise, both proctors and trainees received instructions on how to complete the exercise. Also, proctors were given instructions on how to use the proctoring tool. The use of each proctoring tool was first demonstrated to the proctor by the researcher. Then, the proctor was given up to 2 min to adapt to the new tool. All proctors received the same training for each tool. Finally, proctors were instructed to provide counseling to the trainee on technique, and to correct any technical errors committed. Each dry-lab exercise targeted specific technical skills (e.g. *Endowrist* manipulation, needle driving, knot tying, etc.) that the proctor reinforced when he found appropriate.

As a trainee performed an exercise, the proctor verbally pronounced “mentoring moment” when he determined mentoring was warranted. This was an indication for the trainee to pause and receive verbal instruction or instruction using one of the proctoring tools. The type of instruction and time in seconds were recorded for each mentoring moment.

After each exercise, proctors and trainees completed a standardized questionnaire to evaluate the proctoring tool used [9] (referred to as Exercise Questionnaire). Eight questions regarding the proctoring tool were delivered on a 5-point scale and addressed the ability of the proctoring tool to (1) help delineate anatomic structures, (2) improve surgical/technical skills, (3) improve confidence, (4) allow for safe completion of task, (5) work smoothly, (6) be easy to use, (7) be helpful, and (8) be more helpful than 2D telestration. The 5-point scale was defined with 1 = “Strongly Disagree”, 2 = “Moderately Disagree”, 3 = “Undecided”, 4 = “Moderately Agree” and 5 = “Strongly Agree”. The Exercise Questionnaire has been used in previous research studies as an effective tool to differentiate preferences for various proctoring modalities [9]. At the end of the study, proctors and trainees completed a standardized, post-questionnaire rating the overall effectiveness of each proctoring tool as well as the 3D video quality for the proctor when using ghost tools (all on a 5 point scale) (referred to as Post-Questionnaire). The Post-Questionnaire 5-point scale was defined as 1 = “Least Effective”, 2 = “Moderately Ineffective”, 3 = “Neutral”, 4 = “Moderately Effective”, and 5 = “Most Effective”.

### Analysis

The median and range of the proctor and trainee responses were reported. In addition, the cumulative mentor time, number of instructions provided by the proctor, and average mentor time per instruction were examined and compared across proctoring tool. Some types of instructions were universal across all exercises (e.g., ineffective use of two hands, excessive force, ineffective visualization, etc.) while others were exercise-specific (e.g., dropped ring, missed target, inefficient knot tying technique, etc.). Mann–Whitney *U* tests were used for all pair-wise comparisons of the proctoring tools. Kruskal–Wallis tests were used for group comparisons across all proctoring tools followed by a Dunn’s test to identify which groups, if any, were responsible for the difference.

Chi-square tests were used to evaluate responses to individual questions from the Exercise Questionnaire and Post-Questionnaire. Two categories were created: “agree” and “disagree”. The “agree” category contained responses with values of 4 or 5 (out of 5), and the “disagree” category contained responses with values 1, 2, or 3 (out of 5). Tests of significance compared the categorized proctor and trainee responses to an expected response of 50 % “agree” and 50 % “disagree”.

Finally, Mann–Whitney *U* tests were used to examine the inter-rater reliability of surgeon and non-surgeon proctors and trainees for all proctoring tools on both the Exercise Questionnaire and Post-Questionnaire. A *p* value less than 0.05 was used to determine significance for all statistical tests.

## Results

A total of 26 proctors and twenty-six trainees participated in the study at Keck Medical Center of the University of Southern California (Los Angeles, CA, USA) and Intuitive Surgical (Sunnyvale, CA, USA). Twelve proctors were experienced surgeons and 14 were experienced trainers. Twelve trainees were surgeons in training and 14 trainees were non-surgical subjects inexperienced in robotic surgery. Seven pairs of proctor-trainees were unable to complete all four training exercises due to time constraints. The total number of proctored exercises for each proctoring tool was 23 (2DTele), 20 (3DPointers), 26 (3DHands), and 23 (3DInstruments).

Proctors evaluated all four types of proctoring tools favorably (median responses were ≥3 across all categories from the Exercise Questionnaire; see Table 1). The median proctor response indicated 3DHands and 3DInstruments were more effective than 2DTele (column “Vs2DTele” in Table 1); however, only 3DInstruments showed a significant difference compared to 2DTele (*p* = 0.02). 2DTele was the only proctoring modality that achieved significance for ease of use by proctors (“Easy” in Table 1). The “Easy” score for 3DPointers was particularly low, which was also reported anecdotally by proctors during the study.

**Table 1:** Proctor responses to the exercise questionnaire

Proctor	Anatomy	Surgical	Confident	Safe	Worked	Easy	Helpful	Vs2DTele
2DTele	4 (2–5)	3.5 (1–5)	4 (2–5)	4 (2–5)	4 (2–5)	4 (2–5)*	4 (2–5)	
3DPointers	3 (1–5)	4 (1–5)	3.5 (1–5)	4 (1–5)	4 (1–5)	3 (1–4)	4 (1–5)	3 (1–5)
3DHands	4 (2–5)*	4 (2–5)*	4 (3–5)*	4 (3–5)*	4 (3–5)*	4 (2–5)	4 (2–5)	4.5 (1–5)
3DInstruments	4 (2–5)	4 (3–5)*	4 (3–5)*	4 (2–5)*	4 (2–5)*	4 (2–5)	4 (2–5)*	5 (3–5)*

Responses were on a 5-point scale. Values are reported as median with range in parentheses

An asterisk denotes significant difference between “agree” versus “disagree” responses (*p* < 0.05, Chi-square test)

Trainees also evaluated all four types of proctoring tools favorably (median response ≥3 across all categories from the Exercise Questionnaire; see Table 2). In general, trainee median evaluations were higher than proctor evaluations, but this difference was not significant (*p* > 0.05). In particular, trainees evaluated 3DPointers, 3DHands, and 3DInstruments as more effective than 2DTele, however, only 3DHands (*p* < 0.001) and 3DInstruments (*p* < 0.001) were significantly different. Unlike with proctors, there existed a significant difference for the three ghost tools for ease of use (“Easy”) (3DPointers *p* = 0.03, 3DHands *p* = 0.006, and 3DInstruments *p* < 0.001).

**Table 2:** Trainee responses to the exercise questionnaire

Trainee	Anatomy	Surgical	Confident	Safe	Worked	Easy	Helpful	Vs2DTele
2DTele	3 (1–5)	4 (2–5)	4 (2–5)	4 (2–5)	4 (2–5)	4 (2–5)	4 (3–5)	
3DPointers	3 (2–5)	4 (2–5)*	4 (2–5)	4 (2–5)	4 (2–5)*	4 (2–5)*	4 (1–5)	4 (1–5)
3DHands	4 (2–5)	4 (3–5)*	4 (3–5)*	4.5 (3–5)*	4 (2–5)*	4 (1–5)*	4 (1–5)*	5 (1–5)*
3DInstruments	4 (2–5)	5 (3–5)*	4 (3–5)*	5 (3–5)*	5 (2–5)*	5 (1–5)*	5 (2–5)*	5 (1–5)*

Responses were on a 5-point scale. Values are reported as median with range in parentheses

An asterisk denotes significant difference between “agree” versus “disagree” responses (*p* < 0.05, Chi-square test)

From the Post-Questionnaire, proctors’ overall evaluations of the three types of ghost tools were positive (median responses were ≥3; see first row of Table 3). The overall evaluation of 3DInstruments was significant (*p* = 0.01). In addition, proctors rated 3DInstruments as significantly more effective than 2DTele and 3DPointers (*p* < 0.001, *p* = 0.05, respectively). Similarly, proctors rated 3DHands as significantly more effective than 2DTele (*p* = 0.03). Finally, proctors rated the ability to see the operative field in 3D as more effective than 2D (*p* < 0.001).

**Table 3:** Post-questionnaire results for proctors

Proctors	2DTele	3DPointer	3DHands	3DInstruments
Overall evaluation	3 (1–4)	4 (1–5)	4 (2–5)	4 (3–5)*
Compared to 2DTele (*p* value)		0.3	0.03*	<0.001*
Compared to 3DPointers (*p* value)			0.5	0.05*
Compared to 3DHands (*p* value)				0.07

The overall evaluations are reported as median (range) since responses were on a 5-point scale. The comparisons are reported as *p* values

An asterisk denotes significance (*p* < 0.05, Chi-square test for Likert items comparing “agree” and “disagree” responses within a type of proctoring tool, and Mann–Whitney *U* tests to compare responses between proctoring tools)

Similar to the proctors, trainees’ overall evaluation of the three types of ghost tools from the Post-Questionnaire was positive (median responses were ≥4; see first row of Table 4). The overall evaluation for both 3DHands and 3DInstruments achieved significance (*p* = 0.01, *p* < 0.001, respectively). In addition, trainees rated 3DInstruments as significantly more effective than 2DTele and 3DPointers (*p* < 0.001 for both). Furthermore, trainees rated 3DHands as significantly more effective than 2DTele (*p* = 0.002) and 3DPointers (*p* = 0.01). Based on a comparison of trainee and proctor responses to the Post-Questionnaire, trainees evaluated 3DInstruments and 3DHands as more effective than proctors’ evaluations (*p* = 0.03, *p* = 0.04, respectively).

**Table 4:** Post-questionnaire results for trainees

Trainees	2DTele	3DPointer	3DHands	3DInstruments
Overall evaluation	3 (1–4)	4 (1–5)	4 (2–5)*	4 (3–5)*
Compared to 2DTele (*p* value)		0.2	0.002*	<0.001*
Compared to 3DPointers (*p* value)			0.01*	<0.001*
Compared to 3DHands (*p* value)				0.13

The overall evaluations are reported as median (range) since responses were on a 5-point scale. The comparisons are reported as *p* values

An asterisk denotes significance (*p* < 0.05, Chi-square test for Likert items comparing “agree” and “disagree” responses within a type of proctoring tool, and Mann–Whitney *U* tests to compare responses between proctoring tools)

Additionally, there existed a significant difference across proctoring tools for proctors (*p* = 0.003) and trainees (*p* < 0.001) using a group comparison from Post-Questionnaire responses. For proctors, the mean ranks of 3DInstruments was significantly greater than 2DTele (*p* < 0.05). For trainees, the mean ranks of 3DInstruments and 3DHands were significantly greater than 3DPointers and 2DTele (*p* < 0.05).

**Fig. 2:**
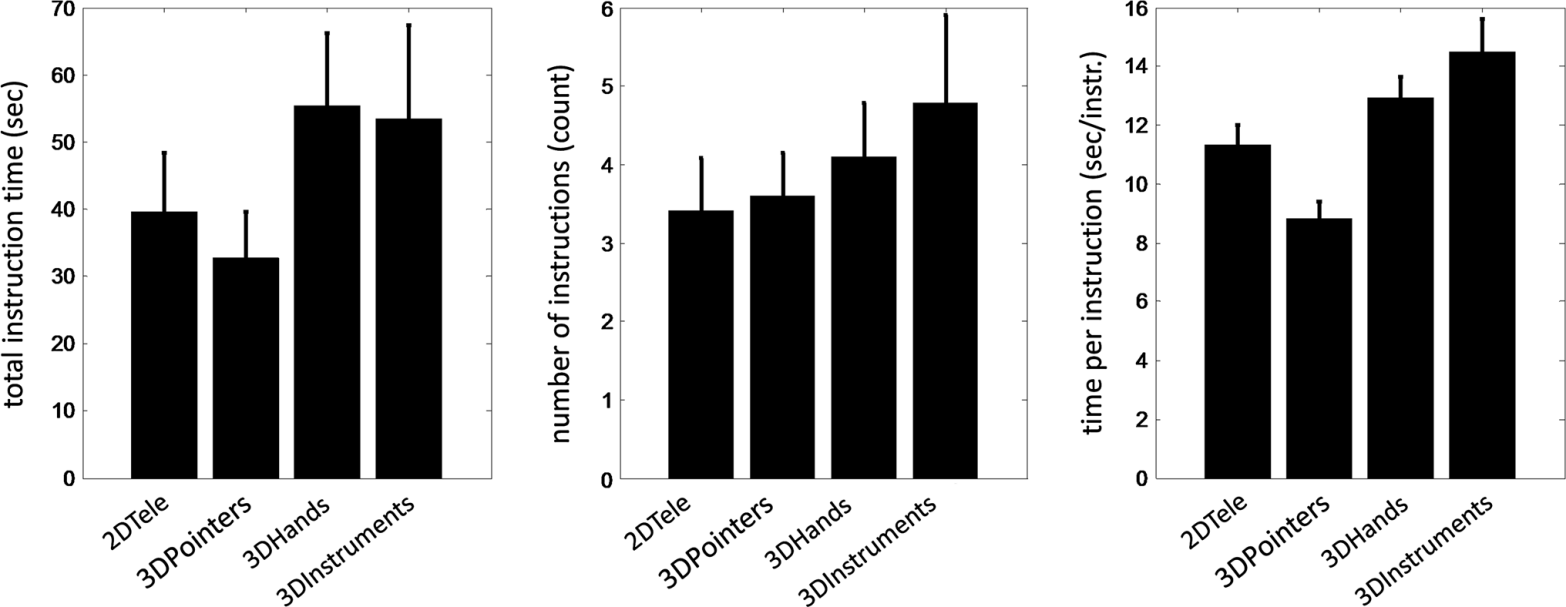
Metrics quantifying proctor-trainee interactions for each proctoring tool (mean with standard *error bar*). Total instruction time (*left*) was the cumulative time a proctor provided instruction to a trainee. Number of instructions (*middle*) was the number of times a proctor provided instructions. Time per instruction (*right*) was the total instruction time normalized by the number of instructions

The cumulative mentor time, number of instructions, and average mentor time per instruction were not significantly different across proctoring tools (*p* = 0.49, *p* = 0.83 and *p* = 0.26, respectively) but trended toward longer mentor times, number of instructions, and mentor time per instruction for 3DHands and 3DInstruments compared to 2DTele and 3DPointers (Fig. 2).

Finally, we compared how surgeon proctors and trainees evaluated ghost tools relative to non-surgeon proctors and trainees given the heterogeneity of the proctor and trainee populations. The only significant difference was that surgeon proctors and surgeon trainees evaluated 2DTele as more effective than non-surgeon proctors and non-surgeon trainees (*p* = 0.03 and *p* = 0.008, respectively) in the Post-Questionnaire.

## Discussion

Proctored cases by an experienced surgeon remain a fundamental component of a new surgeon’s training pathway. During RAMIS, proctors can interact with trainees in novel ways compared to other forms of surgery [21–23]. In this work, we extend these RAMIS proctor-trainee interactions by studying three novel types of proctoring tools called ghost tools (Fig. 1). Ghost tools have two general advantages over conventional proctoring methods; they enabled proctors to see in 3D and to move in 3D with enriched interactions. Indeed, proctors preferred using ghost tools over conventional telestration at the patient side touchscreen (Tables 1, 3), as well as, having a 3D view of the operative field.

However, proctoring technologies impact both the trainee and the proctor and, therefore, careful consideration of both user groups must be made throughout the development process. We illustrate the preferences of these two groups in this study, in particular by the fact that trainees evaluated instruction via all of the ghost tools as easy to accept, whereas proctors evaluated 2D telestration as easier to use than the ghost tools. This could have been mitigated if proctors had more time to acclimate to the ghost tools setup, especially given their familiarity with the da Vinci Surgical System controls (and existing 2D telestration).

Although in-person proctoring will remain essential, there exists a tremendous opportunity for remote proctoring to alleviate geographic and time constraints placed on experienced surgeons serving as proctors [24–28]. In this way, remote proctoring might increase both the number of surgeons proctored and extend the number of cases over which a new surgeon receives some form of expert guidance. The end goal is perhaps better-trained surgeons performing safer surgeries. Future research studies examining how ghost tools may impact the remote proctoring process and the necessary technical specifications (e.g., latency limits [29]) will be needed in order to deliver the most effective interactions between proctors and trainees.

Nevertheless, this study served as an important step to refine the types of interactions between proctors and trainees before moving to a more complex and unstructured environment such as porcine tasks, cadavers, or clinical settings or a remote setup. Although the results of this study are compelling, the utility and performance of ghost tools should be further evaluated on realistic surgical tasks (i.e., tissue dissection, tissue retraction, and anatomy identification). If ghost tools are demonstrated to be efficient and safe in these wet-lab scenarios, clinical testing could be done to determine efficacy in live surgery. Even so, given the results of this study, ghost tools seem to offer advantages during training scenarios as simple as dry-lab tasks that target basic technical skills. Since these sorts of training tasks are commonly performed by new RAMIS surgeons, proctored interactions using ghost tools during similar exercises may help improve the efficiency and effectiveness of surgeon training prior to their first clinical procedures.

A potential limitation with this study was the heterogeneity of proctor and trainee groups. That, along with the small cohorts, could have affected how the ghost tools were evaluated, both from the proctor’s and trainee’s perspectives. However, the only significant difference between groups for both proctors and trainees was how they evaluated 2DTele—surgeons were more favorable of the technology than non-surgeons. One reason might be their familiarity and reliance on two-dimensional telestration for clinical procedures, which non-surgeon proctors and non-surgeon trainees have not experienced.

Another limitation with this study may be that although proctor and trainee preferences of the proctoring tools were elicited, whether their preferences actually translated to practical improvement in proctorship remains unclear. Total mentor time and number of instructions may represent the practical advantage ghost tools have over 2D telestration. However, we did not see a significant difference across proctoring tools with these metrics.

In summary, ghost tools offer compelling improvements over current proctoring tools during RAMIS. They may enable surgeons to move through their learning curves more quickly by providing more effective instruction, and improve patient safety by enabling proctors to more effectively mentor surgeons during clinical procedures. Furthermore, it would be compelling to explore the impact of ghost tools during remote tele-mentored clinical cases and to compare them to existing technologies such as the da Vinci Connect Proctoring System. In the end, additional research is required to continue to understand optimal proctor-trainee interactions.
